# Evidence Suggesting Possible Exposure to Influenza A Virus in Neotropical Bats from Mexico

**DOI:** 10.3390/pathogens14050414

**Published:** 2025-04-25

**Authors:** Brenda Aline Maya-Badillo, Guillermo Orta-Pineda, Gerardo Suzán, Karen Elizabeth Rivera-Rosas, Diego Zavala-Vasco, Adrián Uribe-Jacinto, Andrea Chaves, Alfredo Grande-Cano, René Segura-Velazquez, José Iván Sánchez-Betancourt

**Affiliations:** 1 Laboratorio de Ecología de Enfermedades y Una Salud del Departamento de Etología, Fauna Silvestre y Animales de Laboratorio, Facultad de Medicina Veterinaria y Zootecnia, Universidad Nacional Autónoma de México, Ciudad Universitaria, Ciudad de Mexico 04510, Mexico; mayis.bamb20@gmail.com (B.A.M.-B.); guillermo.orta@senasica.gob.mx (G.O.-P.); gerardosuz@gmail.com (G.S.); 2 Laboratorio de Investigación del Departamento de Medicina y Zootecnia de Cerdos, Facultad de Medicina Veterinaria y Zootecnia, Universidad Nacional Autónoma de México, Ciudad Universitaria, Ciudad de Mexico 04510, Mexico; karen_river24@hotmail.com (K.E.R.-R.); diegozavamvz@gmail.com (D.Z.-V.); 3 Comisión México-Estados Unidos para la Prevención de la Fiebre Aftosa y Otras Enfermedades Exóticas de los Animales, Servicio Nacional de Sanidad, Inocuidad y Calidad Agroalimentaria, Cuajimalpa de Morelos, Ciudad de Mexico 05110, Mexico; 4 Departmento de Ciencias Agropecuariast, Universidad Juárez Autónoma de Tabasco, Villahermosa 86025, Mexico; uribeadrian06@gmail.com; 5 Centro Nacional de Innovaciones Biotecnológicas (CENIBiot), CeNAT, Conare, San José 1174-1200, Costa Rica; andreachaves.biol@gmail.com; 6 Escuela de Biología, Universidad de Costa Rica, San José 11501-206, Costa Rica; 7 Departmento de Secuenciación, Unidad Universitaria de Secuenciación Masiva de DNA-UNAM, Cuernavaca 62210, Mexico; ricardo.grande@ibt.unam.mx; 8 Unidad de Investigación de la Facultad de Medicina Veterinaria y Zootecnia, Universidad Nacional Autónoma de México, Ciudad de Mexico 04510, Mexico; realselab@gmail.com

**Keywords:** *Alphainfluenzavirus*, forest bats, prevalence, southeast Mexico, tropical forests

## Abstract

With the first evidence of the association between bats and influenza A viruses, various studies have begun to emerge to understand this interesting and important association among bats conservation, animal health, and public health. This study aimed to verify the presence of anti-influenza A vipothesrus antibodies, as well as the molecular identification of these viruses in bats distributed in forest fragments located in southeastern Mexico. Blood samples were obtained from 600 bats belonging to 24 different species, using an enzyme immunoassay to detect antibodies against the nucleoprotein antigen of the avian influenza A virus. Likewise, oropharyngeal swabs, rectal swabs and organs were taken for quantitative reverse transcription PCR (qRT-PCR) of these viruses. A total of six bats (1%) tested positive either by serology or molecular methods, not both simultaneously. Although this suggests a very low prevalence of influenza A viruses in Mexican bats, it is the first study to address this association and, following the precautionary principle, we consider it necessary to establish systematic monitoring of the presence of influenza A in bats, since they are known to harbor infectious agents with zoonotic potential. Furthermore, it is possible that the association of influenza A viruses circulating in Latin American bats has an important co-evolutionary component with some bat species with exclusive distribution in the American continent.

## 1. Introduction

Given the major impacts that influenza A viruses (*Alphainfluenzavirus influenzae*) can have on public health, farmers, livelihoods, the poultry industry, and international trade, these viruses have captured increasing attention in the international community [1]. Therefore, identifying the viral reservoirs is urgently needed to better understand the viral evolution and potential cross-species transmissions. Influenza A viruses are known to be present in different domesticated animals (e.g., ducks, chickens, pigs, horses, and dogs). Yet, their presence in wildlife is not as well known, especially in some species-rich and widely distributed taxa, such as bats [2,3].

Bats are known to have multiple associations with several infectious agents because, in general, they have relatively long lifespans, and some species can travel long distances and use anthropogenic landscapes where there may be increased contact (and interaction) between humans, wildlife, and domestic species [2,3]. Bats have been shown to have the potential to host a large proportion of viruses with zoonotic potential [4]. Some infectious agents that bats have been associated with have zoonotic potential, such as Lyssavirus, coronaviruses and members of the influenza A virus [5]. However, the ability of bats to host influenza A viruses and the potential risk of interspecies transmission that this association could trigger remain poorly known.

The great interest in learning more about this virus–bat association arose from recognizing the influenza A virus in fruit bat species in Guatemala and Peru—designated as H17N10 and H18N11 which are subtypes currently considered exclusive to bats [6,7]. In the case of influenza A viruses related to other host species, exposure and infection by virus subtype H9 have been identified in fruit bats from Africa [8,9]. This evidence allows us to hypothesize that some bat species might have ecological and evolutionary conditions that support their participation in the ecology of avian influenza A viruses.

However, there is still a large information gap regarding the ecology of influenza A viruses in bats and their relationship with other hosts. To fill this gap, our study aimed to verify the prevalence of antibodies against influenza A viruses and identify the infection of these viruses in bats sampled in a human-modified tropical rainforest from southeastern Mexico. This information is highly valuable as previous research on the topic has assessed the presence of viruses with molecular detection of viral RNA. For instance, studies carried out in America [5,6,7,10] and Africa [9] have identified bat species mainly infected by the specific subtypes of this group of mammals or through genetic sequencing and have managed to detect an avian subtype. However, the lack of knowledge regarding the development of infection caused by influenza A viruses in bats, and the difficulty of obtaining an adequate amount of blood samples hinders the possibility of detecting individuals exposed to different influenza A subtypes [11]. The use of serological and molecular methods allows us to broaden our understanding of the associations between bat species and influenza A viruses, as well as detect past exposures and infections. Because of this, the purpose of this study was to identify the prevalence of antibodies against influenza A viruses and the molecular prevalence of these viruses in Bats from southeast Mexico distributed in fragmented tropical rainforests.

## 2. Materials and Methods

The fieldwork was authorized by the Secretariat of Environment and Natural Resources (permit number SGPA/DGVS/04241/19) and showed the approval of the Institutional Animal Care and Use Subcommittee with protocol number SICUAE.DC-2021/3-2 of the Postgraduate Program in Animal Production and Health Sciences of the National Autonomous University of Mexico.

**Figure 1:**
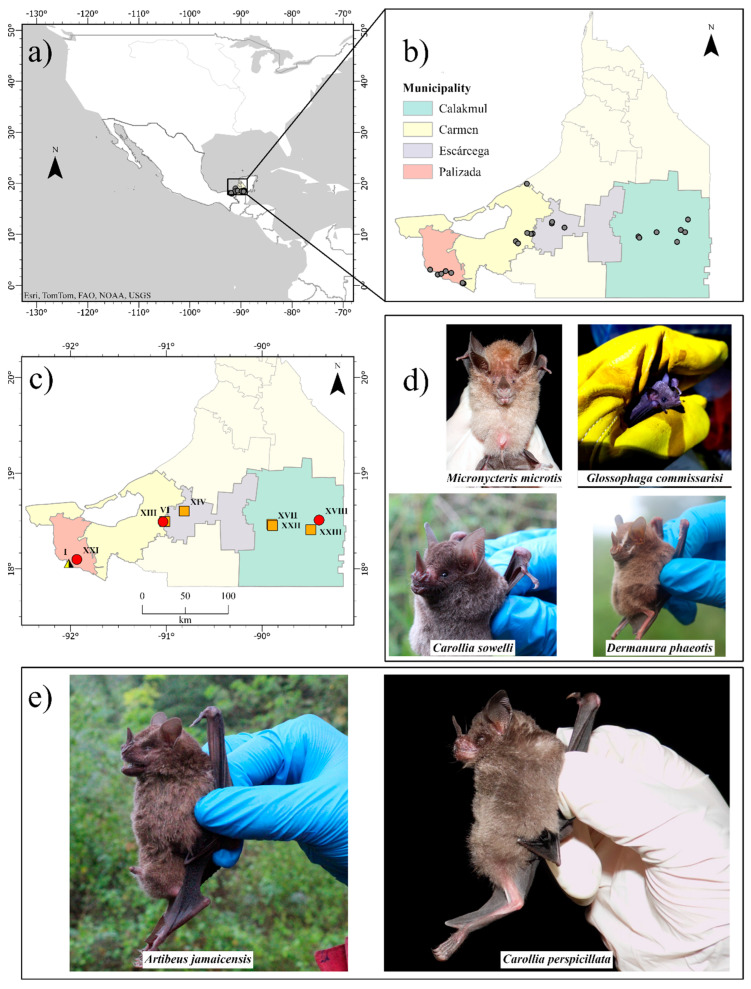
Location of 23 sampling sites in the state of Campeche, Mexico (panels (**a**,**b**)). The six bats that tested positive for influenza A antibodies were captured at four sites in three different municipalities, shown in red (sites I, XIII, XVIII, and XXI). The six sites where the six molecularly positive bats were captured are shown in orange (sites I, VI, XIV, XVII, XXII, and XXIII). Only site I, indicated with a triangle, had seropositive and molecularly positive individuals (panel (**c**)). Positive species are shown in panels (**d**,**e**); the latter panel shows the two species with the most positive individuals.

This study was carried out from April 2021 to April 2022 in 23 forest sites located within 23 rainforest fragments from the state of Campeche, Mexico (Figure 1a,b). Within each forest site, we placed six 12 m × 2.5 m mist nets covering as much area as possible and recorded all bats captured during 14 h/net per site for two consecutive nights. The networks were opened at sunset and seven hours later they were closed. Once captured, each individual was collected in a separate cloth bag for later identification and sampling. The identification of the bat species was carried out through identification guides and trained personnel. Bats were released after sampling and only some were selected for euthanasia and necropsy to meet other project objectives. The selected individuals were only individuals belonging to species that are not in danger of extinction or threatened, that is, they are not found in any of the risk categories and are in the category of least concern in Mexican standards and their populations are also stable. In addition to the species, only adult females or males and juvenile females or males were selected. No pregnant or lactating females were selected.

The blood samples were collected from the brachial vein, not exceeding more than 10% of their body weight, using filter paper, and then the samples were stored at −20 °C until processed [12]. Individuals weighing 10 g as well as pregnant or lactating females or individuals in any physiological state of protected species were not sampled so as not to compromise their lives. Filter paper strips were eluted in 400 µL of phosphate-buffered saline in 1.5 mL microcentrifuge tubes at 4 °C for 24 h. Afterward, the samples underwent serum heat inactivation at 56 °C for 30 min. Finally, the supernatant was obtained through centrifugation at 3000 rpm for five minutes. Therefore, the serum was obtained with a 1:10 dilution [13]. To identify sera containing influenza A antibodies, we used an enzymatic immunoassay to detect antibodies for avian influenza A virus nucleoprotein antigen (IDEXX Influenza Virus Ab Test Kit). The assay was performed in 96-well plates coated with the viral antigen of influenza A viruses. After incubation of the sample analyzed in the coated wells, the specific antibody against influenza A viruses forms a complex with the antigen. Subsequently, unbound material was removed, and an anti-influenza A monoclonal antibody-enzyme conjugate was added to the wells. Since there are no antibodies against the NP of the influenza A viruses, the conjugate reacts directly with the antigen of the influenza A viruses present on the plate. Conversely, if there are antibodies against the NP of influenza A viruses present in the sample, the anti-influenza A conjugate will not be able to bind to the antigen. The unbound conjugate is removed by washing and an enzyme substrate is added. The color that appears is inversely proportional to the amount of anti-influenza A antibodies in the sample analyzed. To identify seropositive individuals, we considered those with less than 0.7 nm sera positive [14].

The positive samples underwent a microneutralization assay for influenza subtypes H1N1, H5N1, H7N3, and H9N2 of the avian origin with an ELISA-based endpoint evaluation as described in the protocol available from the World Health Organization (WHO) [15]. Virus viability was assessed with MDCK cells in 96-well plates using ELISA to identify positive culture wells with infected cells. Subsequently, in the 96-well plate, it was mixed with the serum dilution to be analyzed and left to incubate for one hour at 37 °C and then a suspension of MDCK cells was added to each well. At 22 h post-infection, the cells were fixed with acetone. The virus infection in cells in each well is assessed by ELISA as described in the WHO protocol. The neutralization titer will be considered as the reciprocal of the highest serum dilution.

We also took samples of oropharyngeal and rectal scrapings through swabs that were transported in cryotubes with MEM medium in liquid nitrogen. The samples were stored deep frozen at −70 °C until processing. Similarly, samples were taken from the trachea, lungs and intestines of some species of bats whose populations are stable. Euthanasia was performed using an inhalation anesthesia capsule specifically designed for bats where Isoflurane was used at a concentration of 2 to 5% to bring the individuals to a deep anesthetic plane where they are desensitized [16,17]. They were subsequently administered an overdose of ketamine (10 mg/kg of body weight) for euthanasia [18]. Once the death of the individuals was confirmed, a necropsy was performed and samples were taken from the trachea, lung, and small and large intestine (Supplementary Video S1) [10]. These samples were transported in 2 mL cryotubes in liquid nitrogen and stored in the same way as the scraping samples through swabs. Subsequently, the viral RNA was extracted from the oropharyngeal, rectal scraping samples and the organs using the commercial kit “QIAamp^®^ Viral RNA (Qiagen)” and using linear acrylamide as a carrier, following the specifications recommended by the manufacturer. After this, the qRT-PCR (Real-Time Polymerase Chain Reaction) test was performed aimed at the amplification and detection of the M protein (Matrix) with the use of the commercial kit “VetMAX-Gold AIV Detection Kit” (Life Technologies) following the recommendations established by the manufacturer. Positive samples were considered those that obtained a C_T_ value less than 38, suspicious samples were those that obtained a value of 38 to 40, and negative samples were those that did not obtain any value.

Samples that tested positive were processed for whole genome amplification and sequencing. Genome amplification was performed through a multi-segment RT-PCR reaction using universal oligonucleotides. The oligonucleotides used are described in [19,20] and for library construction, following in detail the protocol described in https://www.protocols.io/view/a-sequencing-and-subtyping-protocol-for-influenza-n2bvj8mrxgk5/v1, accessed on 23 April 2025. The RT-PCR reaction products were purified and concentrated using Ampure XP Beads (Beckman, Coulter). The amplified fragments were verified as 1% agarose gel. The amplification products were tagged using the EBLTS (beads) of the COVIDSeq Assay Kit to finally amplify and label the tagged fragments using the Nextera XT Index Kit v2 as specified in the protocol. The sequence was performed on an Illumina NextSeq 500 device in a configuration of 2 × 150 cycles and 200 K reads per sample.

## 3. Results

**Figure 2:**
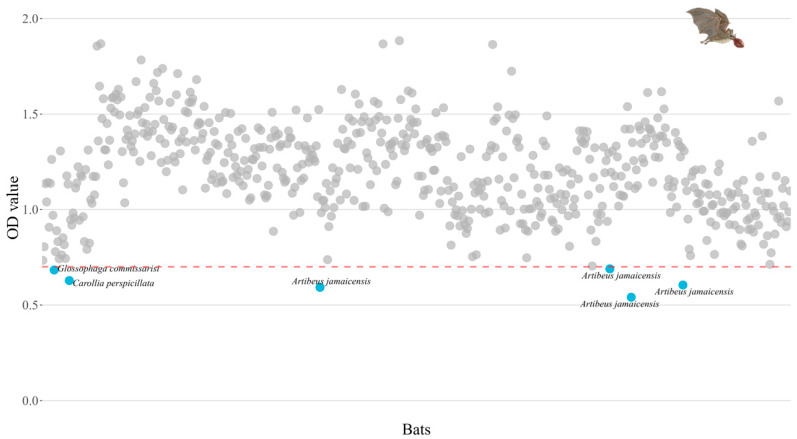
Serological evidence of influenza A virus antibodies in bats from Campeche, Mexico. Blue dots represent the sera that obtained values below the cutoff value (0.7 nm) represented by the red dotted line; these sera are considered positive for the presence of influenza A virus antibodies.

We obtained samples from 600 bats belonging to 24 species and four families. Phyllostomidae was by far the most abundant family, with *Artibeus jamaicensis* (36% of captured individuals), *Dermanura phaeotis* (17%), and *Artibeus lituratus* (13%), being the most abundant species (Supplementary Table S1). Only six individuals from three species (i.e., *Artibeus jamaicensis*, *n* = four individuals, of which four are adult females, one pregnant, one lactating and two post-lactating; *Carollia perspicilliata*, *n* = one, adult female; and *Glossophaga commisarissi*, *n* = one, pregnant female) presented serological evidence of influenza A antibodies, but it was not possible to identify the subtype to which they were exposed because we obtained negative results in microneutralization for the H1N1, H5N1, H7N3, and H9N2 subtypes of avian origin. *Artibeus jamaicensis* and *Carollia perspicilliata* are frugivorous species, and *Glossophaga commisarissi* is nectarivorous. These individuals were captured in the municipalities of Palizada, Carmen, and Calakmul (Figure 1c–e and Figure 2). Similarly, we identified six RT-PCR positive individuals from five different species (i.e., *Artibeus jamaicensis*, *n* = two individuals, of which one is a juvenile male and the other is an adult male; *Carollia sowelli*, *n* = one, adult male; *Carollia perspicillata*, *n* = one, adult male; *Dermanura phaeotis*, *n* = one, juvenile female; and *Micronycteris microtis*, *n* = one, adult female. The quantification of the cycle threshold (Ct) ranged between 35.45 and 37.9 (Figure 1c–e; Table 1). The RT-PCR positive samples correspond to oropharyngeal and rectal swab samples. The individuals with these positive samples were captured in Palizada, Carmen, Escárcega, and Calakmul. No positive results were obtained from the tracheas, lungs and intestines of the individuals selected for necropsy (*n* = 270 individuals out of 600). The RT-PCR positive samples were amplified for all eight segments, but it was not possible to assemble the genome and reach a conclusive result.

## 4. Discussion

Bats have been associated with a long list of infectious agents mainly due to their resistance to infections and their great adaptive and co-evolutionary capacity. In the Americas, various species of neotropical bats have been primarily associated with RNA viruses, with notable findings in the families Phyllostomidae, Moormopidae, Molossidae, and Vespertilionidae [21]. Our findings suggest possible exposure and infection of influenza A viruses in neotropical bats associated with tropical forests and human-modified landscapes in southeastern Mexico.

Only six out of 600 bats (1%) tested positive by serology and another six individuals (1%) tested positive by RT-PCR, indicating a very low seroprevalence and molecular prevalence of influenza A viruses in the study region. However, as these are not isolated cases, our findings add to the growing evidence indicating that some bat species may participate in the ecology of influenza A viruses [5,6,7,8,9,10]. Although sequencing data were not obtained due to the low RNA concentration in the samples, the qRT-PCR results—despite high Ct values—suggest possible exposure and infection. Each positive result was confirmed through repeated testing to ensure reproducibility. Nonetheless, in comparison to other hosts of these viruses such as wild birds, there is still ignorance, in most parts of the world, about the participation of wild mammals, such as bats, in transmission cycles, particularly in the infection and exposure to influenza A viruses. The detection of viral RNA in oral swabs but not in lung tissue may indicate early infection, as initial influenza A virus replication typically occurs in the upper respiratory mucosa before disseminating to the lower respiratory tract. This pattern could also reflect different stages of infection: while RT-PCR positivity from oral swabs may represent an early or active infection, ELISA positivity is more likely to indicate previous exposure and the presence of an established immune response.

This information supports the hypothesis of a possible co-evolutionary association between the Phyllostomidae family found only in America and the H17N10 and H18N11 subtypes. This is why assays with a higher degree of specificity may reduce the coverage of positive individuals by not having the specific subtypes to identify exposure to and infection with influenza viruses in bats. In this study, we had the limitation of not having the H17 and H18 subtypes and the rest of the H2–H4, H6, and H8–H16 subtypes to carry out the microneutralization tests and obtain more specific information about the association of the bats in our study with different subtypes of influenza A viruses. Likewise, it is important to recognize the limitations of the ELISA assay and to overtime generate panels of relevant HI viral antigens that cover the exposure of bats to influenza A viruses in a regional and global context. The inability to sequence the samples limits definitive viral identification; however, the amplification of the eight gene segments via qRT-PCR reinforces the possibility of genuine detection despite this constraint.

However, despite the limitations, our molecular results demonstrate molecular evidence of influenza A virus infection in 6 bats of 5 of the 24 species captured in Campeche. It has been shown that the detection of influenza A viruses in bats is very low, for example, Tong et al. (2012) evidenced for the first time the presence of H17N10 in fruit bats of the species *Sturnira lilium* in Guatemala with a positivity frequency of 0.94% (3 positive individuals out of 316 total bats) [6]. Similarly, in 2013 the H18N11 virus was identified in *Artibeus planirostris* from Peru, with a positivity frequency of 0.1% [7]. The same has happened in more recent years, for example, in Brazil, in 2019, an H18N11 was demonstrated for the first time in bats of the species *Artibeus lituratus* with a frequency of 0.4%, that is, 2 individuals out of 533 bats [10]. Under these characteristics, our results show, for the first time in Mexico, serological evidence about the possible exposure of Phyllostomidae bats to influenza A viruses in the neotropical region. However, the number of studies on the association between bats and the influenza A virus is very low compared to the number of studies on other species. This panorama reinforces the importance of exploring this association in greater depth with new research, especially in fruit bats due to the coincidence reported so far.

The findings of this study indicate that the competitive ELISA that we used can be used for seroepidemiological studies of influenza A in species that have been little explored and have difficulty obtaining quality samples of because the objective of the test is to detect the presence of nucleoprotein antibodies of the anti-influenza A virus cross-reacting with influenza A viruses and this region is highly conserved. In such a way that the detection coverage of positive individuals is increased, which on many occasions cannot be detected by other specific tests such as hemagglutination inhibition (HI) due to the specificity of the viral subtype. In this sense, the samples positive to the ELISA were not positive to the microneutralization assay directed at the avian H1N1, H5N1, H7N3, and H9N2 subtypes due to the specificity of the test and the subtypes used. Although these subtypes are of great relevance in the epidemiological context of influenza A viruses due to their impact on animal health and public health, as is the case with the H1N1, H5N1, and H7N3 subtypes, we still do not have sufficient knowledge to determine the participation and relationship of these subtypes with neotropical bats. Likewise, although the H9N2 subtype has already been reported in other parts of the world in Old World bats [9], in our study we did not report seropositivity for this subtype, which coincides with what has been reported up to this point for the American continent.

Frugivorous bats, such as those from the *Artibeus* genus, can participate in the ecology of influenza A viruses. As mentioned, the presence of influenza A viruses in bats was first confirmed in *Sturnira lilium* and *Artibeus planirostris*, particularly the H17N10 and H18N11 subtypes in Guatemala and Peru, respectively [6,7]. Some years later, in Brazil, the isolation and sequencing of the H18N11 virus were reported in the intestines of two *Artibeus lituratus* individuals from different places [10]. In Colombia, there are also reports of bat samples, *Carollia* and *Artibeus* in particular, that tested positive for influenza A viruses [5]. Thus, phyllostomid bats are expected to be positive for influenza A viruses, which allows us to hypothesize that it is probable that, besides being exposed, the bats sampled in our research can get infected with the subtypes reported in other parts of America, such as H17N10 and H18N11, as well as avian subtypes as has happened in other parts of the world [6,7,9,10]. Based on the findings reported in American studies, we could suggest a co-evolutive association between the Phyllostomidae family found only in this continent and the H17N10 and H18N11 subtypes as they have not been reported in other parts of the world.

The relatively high prevalence of influenza A viruses in *Artibeus jamaicensis* may be related to the dominance of this bat species in the study region. Bats from the *Artibeus* genus tend to be abundant in Neotropical rainforests [22], and *A. jamaicensis* was the most abundant species in our survey. *A. jamaicensis* is considered an abundant and proportionally dominant species in some communities [23], and this could lead them to be exposed to a higher degree of contact with other bats and other species. In addition to abundance in ecosystems, the gregarious habits of bats that tested positive could make them prone to greater exposure and possible infection with influenza A viruses, along with other infectious agents. The habit of forming large colonies to rest in caves or other shelters, as well as their social structure based on polygyny and the formation of harems of between four to 18 females, their offspring, one or two dominant males that actively defend the harem from other males [23], fission–fusion behavior and longevity, increases the probability of interspecific contact with bats from southeastern Mexico [24].

All the species that showed evidence of infection by influenza A virus belong to the family Phyllostomidae, where 98% of the species samples come from this family. This may be since members of the family Phyllostomidae show evidence of interspecific attraction in mixed-species roosting groups of bats in natural roosts, particularly in the case of species of the genus *Carollia* and *Glossophaga* [25]. A documented example is the co-existence of bats of the genus *Artibeus* with bats of the genus *Carollia* [26]. This co-existence is facilitated by the abundance of food resources that are part of the diet of these species, as well as by the morphological differences that allow differentiation of foraging behavior about fruit consumption, and by the structural characteristics of the vegetation of this type of neotropical forests. These dietary preferences and anatomical differences suggest that other resources such as a preference to rest in caves or certain mechanisms such as predation, parasitosis or the dynamics of certain infectious agents explain their ability to co-exist. The availability of areas within forests and fruits used by these species is so great that there is little need for ecological segregation into different niches [26]. This in turn favors greater interaction between individuals of different species that belong to the same trophic guild. Therefore, it is not surprising that in our study there are positive individuals of other species that are not of the genus *Artibeus*, as is the case of *Carollia*, *Dermanura*, *Glossophaga,* and *Micronycteris* because these species co-exist in the neotropical forests of southeastern Mexico due to the abundance of resources obtained from these diverse forests with great biological complexity. This co-existence may promote the transmission of influenza A viruses between bats of different species that co-exist and share ecological niches within Neotropical forests and that, in addition, various species have closer phylogenetic relationships that allow them to be infected by the same viruses, as has been demonstrated in other studies [27].

Understanding the ecoepidemiology of influenza A viruses among bats and between bats and various species is then the challenge of future research to assess the consequences on animal and public health and to determine possible taxonomic jumps of influenza A viruses. Recently, the position proposed by authors such as Ciminski et al., 2019, is that influenza A viruses detected in bats that are limited to interspecies transmission between bats has been weakened [28]. Experimental conditions have shown that H18N11 subtype viral particles can infect human leukocytes and replicate them easily in human macrophages [29]. Other studies have revealed that bats could play an important role in the ecology of avian influenza A viruses, as epithelial cells in their lungs are susceptible to infection by certain avian-origin viruses and show evidence of avian and human influenza virus compatible sialic acid receptors [30,31]. In bats from Egypt, an influenza A H9N2 virus, phylogenetically distinct from viruses detected in birds, was successfully isolated and it is considered a virus that recently diverged from birds and effectively adapted to Egyptian bats [9,32]. In addition, there is growing information about their association with a wide variety of infectious agents which can potentially generate serious animal and public health consequences [2].

## 5. Conclusions

This study provides the first findings on the potential interaction and infection of Neotropical bats with influenza A viruses and suggests a potential critical list for the target bat species in the viral ecology of these viruses in southeast Mexico. It is necessary to open new research questions and hypotheses to explore the capacity that Mexican bats must harbor different subtypes of influenza A viruses and to understand their participation in the ecology of influenza A viruses in fragmented tropical forests. Because bats harbor a significantly high proportion of viruses with zoonotic potential, it is essential to carry out serological studies in other areas and the molecular subtyping to broaden substantially our understanding of the origin of influenza A viruses in bats and their ability to participate in the transmission cycles of these viruses. With time, it is possible to generate knowledge as extensive as that which exists for other wild hosts, mainly in bats distributed in tropical ecosystems affected by human activities.

## Figures and Tables

**Table 1:** Ct values obtained in bat samples from southeastern Mexico were positive for influenza A virus.

Bats	Ct	Sample
*Artibeus jamaicensis*	36.25	Oropharyngeal swab
*Artibeus jamaicensis*	37.78	Oropharyngeal swab
*Carollia sowelli*	37.9	Oropharyngeal swab
*Carollia perspicillata*	36.61	Oropharyngeal swab
*Dermanura phaeotis*	33.92	Oropharyngeal swab
*Micronycteris microtis*	37.55	Rectal swab

## Data Availability

The data related to this experimental study are available on request from the corresponding author.

## Supplementary Materials

The following supporting information can be downloaded at: https://www.mdpi.com/article/10.3390/pathogens14050414/s1, Supplementary Table S1. Abundance and bats species captured for sampling. Supplementary Video S1. Taking organ samples at a necropsy of selected individuals.
