# Supplementary material for: Evidence Suggesting Possible Exposure to Influenza A Virus in Neotropical Bats from Mexico
Source: Pathogens. 2025 Apr 25;14(5):414. doi: 10.3390/pathogens14050414 (PMC12114349; doi:10.3390/pathogens14050414)
Supplement: Supplementary file 1 [file pathogens-14-00414-s001.zip › pathogens-3576132-supplementary.pdf]

Supplementary Information

Supplementary Table S1. Abundance and bats species captured for sampling.

Family	Subfamily	Specie	Abundance
Phyllostomidae	Stenodermatinae	*Artibeus jamaicensis*	216
Phyllostomidae	Stenodermatinae	*Artibeus lituratus*	77
Phyllostomidae	Carolliinae	*Carollia perspicillata*	34
Phyllostomidae	Carolliinae	*Carollia sowelli*	30
Phyllostomidae	Stenodermatinae	*Centurio senex*	2
Phyllostomidae	Stenodermatinae	*Chiroderma salvini*	1
Phyllostomidae	Stenodermatinae	*Chiroderma villosum*	2
Phyllostomidae	Stenodermatinae	*Dermanura phaeotis*	102
Phyllostomidae	Stenodermatinae	*Dermanura watsoni*	11
Phyllostomidae	Desmodontinae	*Desmodus rotundus*	28
Vespertilionidae	Vespertilioninae	*Eptesicus furinalis*	1
Phyllostomidae	Glossophaginae	*Glossophaga commissarisi*	32
Phyllostomidae	Glossophaginae	*Glossophaga soricina*	4
Phyllostomidae	Phyllostominae	*Lophostoma evotis*	1
Phyllostomidae	Phyllostominae	*Micronycteris microtis*	1
Phyllostomidae	Phyllostominae	*Mimon cozumelae*	1
Vespertilionidae	Myotinae	*Myotis elegans*	1
Natalidae	Vespertilionoidea	*Natalus mexicanus*	2
Phyllostomidae	Phyllostominae	*Phyllostomus discolor*	3
Phyllostomidae	Stenodermatinae	*Platyrrhinus helleri*	1
Mormoopidae	Noctilionoidea	*Pteronotus parnellii*	3
Phyllostomidae	Stenodermatinae	*Sturnira hondurensis*	29
Phyllostomidae	Stenodermatinae	*Sturnira parvidens*	15
Phyllostomidae	Stenodermatinae	*Uroderma bilobatum*	3

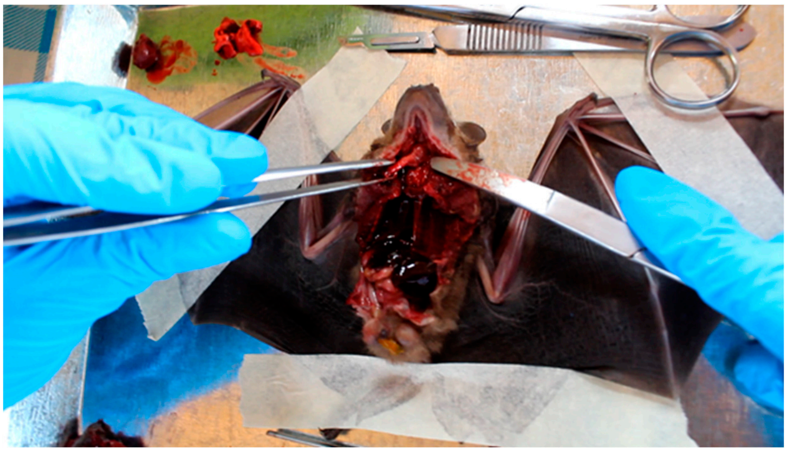

Supplementary Video S1. Taking organ samples at a necropsy of selected individuals (see attached file).
